# Data on the identity and myristoylation state of recombinant, purified hippocalcin

**DOI:** 10.1016/j.dib.2016.04.024

**Published:** 2016-05-20

**Authors:** Anuradha Krishnan, Jeffrey Viviano, Yaroslav Morozov, Venkat Venkataraman

**Affiliations:** a Graduate School of Biomedical Sciences, USA; b School of Osteopathic Medicine Rowan University, Stratford, NJ 08084, USA

**Keywords:** Hippocalcin, Calcium, NCS proteins, MALDI-TOF, ISD sequencing, Myristoylation

## Abstract

In this data article we report on the purity and post translation modification of bacterially expressed and purified recombinant hippocalcin (HPCA): a member of the neuronal calcium sensor protein family, whose functions are regulated by calcium. MALDI-TOF in source decay (ISD) analysis was used to identify both the myristoylated or non-myristoylated forms of the protein. MALDI-TOF ISD data on the identity of the protein, amino acid sequence and myristoylation efficiency are provided. This data relates to the article “Single-Column Purification of the Tag-free, Recombinant Form of the Neuronal Calcium Sensor Protein, Hippocalcin Expressed in *Eschericia coli*” [1].

**Specifications Table**

**Table:** 

Subject area	*Biology*
More specific subject area	*Protein Identification*
Type of data	*Table, figure*
How data was acquired	*In Set Decay Mass Spectrometry (MALDI-TOF ISD), DNA Sequencing*
Data format	*Analyzed*
Experimental factors	*For MALDI-TOF ISD, Standard protocols were used*
Experimental features	*Purified protein was analyzed by MALDI-TOF ISD*
Data source location	*Stratford, New Jersey, USA*
Data accessibility	*Data contained within this article*

Value of the data

• Confirms the identity of the bacterially expressed HPCA purified in a single step.
• Demonstrates the ability to properly myristoylate the bacterially expressed HPCA with high efficiency
• Identifies the first amino acid residue of expressed HPCA
• Provides a benchmark approach to characterizing critical aspects such as myristoylation in bacterially expressed neuronal calcium sensor proteins in particular and modified proteins in general.

## 1 Data

**Fig. 1:**
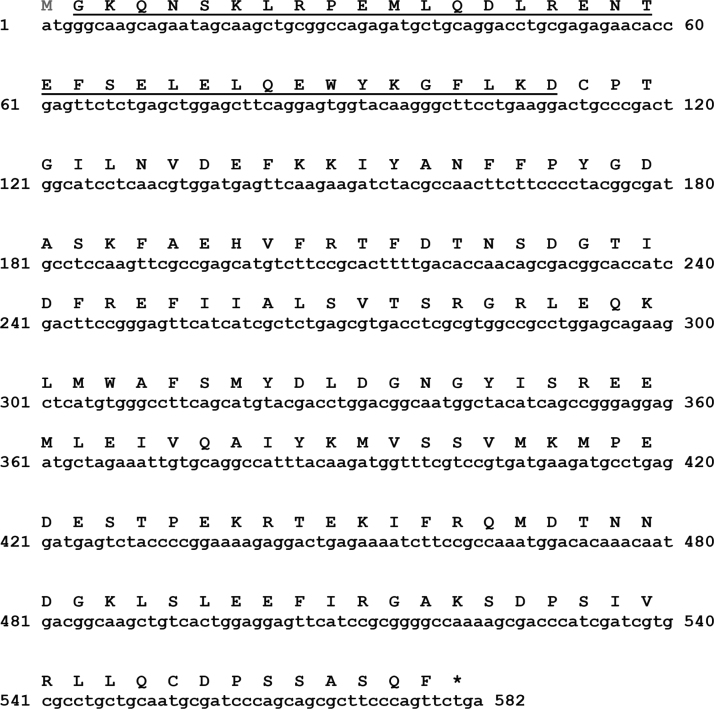
Sequence of expressed HPCA.

Purified HPCA was analyzed through mass spectrometry. MALDI-TOF ISD analyses were independently carried out with the myristoylated and non-myristoylated forms of HPCA. Table 1 displays the sequence of the first 8 fragments identified by ISD. The difference between non-myristoylated and myristoylated forms, as expected, is 210 Da. Data presented in Fig. 1 confirms the identity of the expressed protein, derived from the cDNA sequence as well as through MALDI-TOF ISD (underlined sequence) as HPCA [2], [3]. Together, the data demonstrate the loss of the first methionine (in grey; Fig. 1) in the purified protein.

## 2 Experimental design, materials and methods

HPCA was purified as previously described [1]. Five μg of myristoylated or non-myristoylated HPCA was desalted using C4 ZipTip (Millipore Inc.). The sample was then mixed 1:2 with saturated 1,5-diaminonaphthalene in 50% acetonitrile and 0.1% TFA in water and spotted on the MALDI target plate. In-source decay (ISD) data was collected using Bruker MicroFlex LFR MALDI-TOF in positive linear mode. Mass range was set from 1000 to 7000 Da and the pulse ion extraction was set at 240 ns. ISD spectra were analyzed with Flex Analysis software (Bruker).

Rat HPCA coding region was amplified by PCR and inserted into the bacterial expression vector pET 21d between NcoI and HindIII sites. Sequencing of the construct was performed in both directions (GeneWiz Inc.).

## Figures and Tables

**Table 1:** N-terminal fragments generated from myristoylated and non-myristoylated HPCA.

Fragment	Molecular Weight		
	**Myr**^**−**^	**Myr**^**+**^	**Difference**
GKQNSKLR*P*	1030.0	1240.5	210.5
GKQNSKLRP*E*	1159.9	1369.9	210.0
GKQNSKLRPE*M*	1291.1	1501.4	210.3
GKQNSKLRPEM*L*	1404.3	1614.2	209.9
GKQNSKLRPEML*Q*	1533.5	1742.3	208.8
GKQNSKLRPEMLQ*D*	1647.7	1857.5	209.8
GKQNSKLRPEMLQD*L*	1760.7	1970.0	209.3
GKQNSKLRPEMLQDL*R*	1916.9	2126.6	209.7
